# Thrombosis With Thrombocytopenia and Post-COVID-Vaccination Syndrome With Anti-G-Protein-Coupled Receptor (GPCR) Antibodies Treated With Therapeutic Plasma Exchange

**DOI:** 10.7759/cureus.60019

**Published:** 2024-05-10

**Authors:** Mauro Mantovani, Romano Grossi, Giuseppe Di Fede, Paolo Bellavite

**Affiliations:** 1 Laboratory, Istituto di Medicina Biologica, Milan, ITA; 2 Nephrology and Dialysis, S.M. Goretti Hospital, Latina, ITA; 3 Preventive Medicine, Istituto di Medicina Biologica, Milan, ITA; 4 Medicine, University of Verona, Verona, ITA

**Keywords:** autoimmunity, therapeutic plasma exchange, venous thrombosis, long post-covid vaccination syndrome, covid-19, adverse events following immunization

## Abstract

We present the case of a female who developed cerebral venous thrombosis with thrombocytopenia after inoculation with the anti-coronavirus disease 2019 (COVID-19) Vaxzevria vaccine, followed by splanchnic thrombosis and diffuse hemorrhages. Despite receiving treatment, the complications increased, and hence therapeutic plasma exchange (TPE) was attempted, leading to laboratory and clinical improvements and discharge after a period of intensive care. Almost two years after the first episode, in the interim of which the patient complained of only minor symptoms such as asthenia and difficulty concentrating, she developed an epileptic syndrome that required neurological treatment. In addition, her fatigue and difficulty concentrating worsened and other serious symptoms of dysautonomia appeared, such as trembling of her right arm, loss of stability, and postural orthostatic tachycardia. As serum analysis revealed a significant number of alterations in autoantibodies against various G-protein-coupled receptors (GPCRs) and RAS-related proteins, two further TPEs were performed, resulting in rapid and sustained clinical improvement. This report highlights the role of the different types of autoantibodies produced in response to anti-COVID-19 vaccination, which can have functional, regulatory, and possibly pathogenic effects on the vascular and nervous systems.

## Introduction

Reactions to anti-coronavirus disease 2019 (COVID-19) vaccines affect various organs, with varying severity and duration among patients. The earliest serious adverse events detected include coagulation disorders with venous thrombosis, especially in the case of adenovirus-based vaccinations [1]. Furthermore, it is becoming apparent that vaccinations can also be followed by a subacute or chronic pathological condition, the so-called long post-COVID vaccination syndrome (LPCVS), characterized by general fatigue, muscle and osteoarticular pain, dyspnea, numbness in the extremities, orthostatic tachycardia, hypertension, dyspnea, insomnia, anxiety, dizziness, and neurological and neuropsychiatric disorders [2-4]. Many signs and symptoms of this condition are similar to those of "post-acute COVID-19 syndrome" (PACS) reported in 10-20% of patients [5], suggesting at least a partially common pathogenesis [6]. These diseases often affect the cardiovascular and neurological systems, can impair the quality of life of patients, and are difficult to treat.

In COVID-19, as in many infectious diseases, complications of an autoimmune nature can arise with a prolonged course, particularly associated with the severity of the disease [7-9]. Autoimmune mechanisms have also been implicated in the adverse sequelae of vaccinations [10-12]. In this report, we discuss the case of a patient who suffered initially from acute thrombotic syndrome a few days after the first dose of vaccine, followed by LPCVS, which was marked by a significant change in antibodies against receptors of the autonomic nervous system; the patient achieved full remission of both conditions after plasmapheresis treatment.

## Case presentation

**Figure 1:**
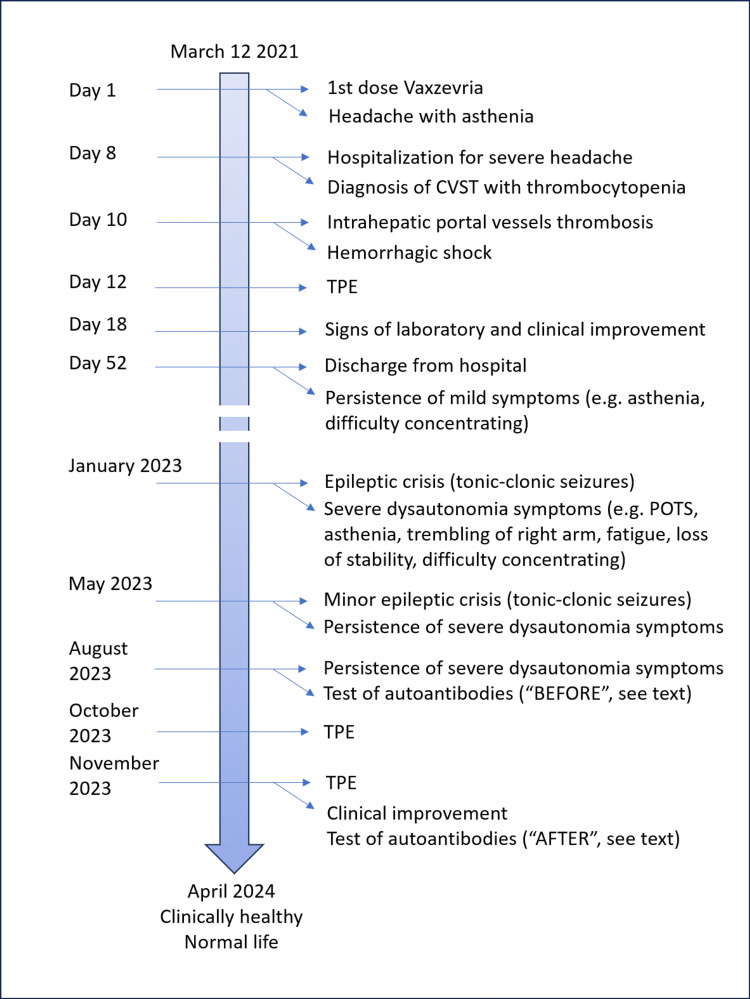
Timeline of the presented case report CVST: cerebral venous sinus thrombosis; POTS: postural orthostatic tachycardia; TPE: therapeutic plasma exchange

Figure 1 depicts the main events/milestones in the clinical history of the presented case.

The patient was a 37-year-old female without any medical history. In the week before receiving her COVID-19 vaccination, she underwent routine blood tests, which showed completely normal Hb, platelets, and coagulation parameters.

She received the first dose of the anti-severe acute respiratory syndrome coronavirus 2 (SARS-CoV-2) vaccine Vaxzevria (ChAdOx1-S) on 12 March 2021; in the evening of the same day, she complained of chills without fever and, in the following days, of headache with asthenia, which was treated with paracetamol. On 19 March 2021, she presented with severe headache refractory to treatment and was admitted to the hospital ("S. Maria Goretti" in Latina, I). The following day, angio-CT and MRI of the brain showed a thrombosis of the cerebral venous sinuses. The platelets were 44,000, D-dimer >80, antithrombin III (ATIII) 75.80, while the fibrinogen level was 178 mg/dl, lower than the day before (281 mg/dl). She was started on enoxaparin sodium 4000 IU x 2 and prednisone PDN 1 mg/kg. Thrombophilia screening revealed the presence of a homozygous MTHFR gene mutation and a slight increase in lupus anticoagulant; all other autoantibodies, including anti-PF-4 Ab, were negative.

On 21 March 2021, following the worsening of abdominal pain symptoms, an abdominal CT scan was performed, which showed thrombosis of the intrahepatic portal vein. This was followed by severe endo-abdominal hemorrhage and hemorrhagic shock requiring blood transfusion. At that time, the platelet count was 18,000, fibrinogen 72 mg%, ATIII 60.90%, and D-dimer 26.93 mg/l.

On 23 March 2021, after a multidisciplinary discussion focusing on restoring immune system balance, the patient underwent therapeutic plasma exchange (TPE). An exchange of ~30-40 ml/kg was performed with a solution of 4% albumin in physiological saline, amounting to a total of ~1800-2000 ml, corresponding to a total blood volume of ~0.70-0.75%. After TPE, the platelet count was 23,000 (rising to 67,000 after platelet pool infusion), fibrinogen level was 143 mg%, ATIII 85.90%, and D-dimer 17.02 mg/l. The following day, as the severe headache persisted with visual disturbances, a new brain CT scan was performed, which revealed a large cerebral venous hemorrhage in the left parietal-temporal-occipital area, with associated large amounts of perilesional edema.

Over time, the platelets and Hb gradually stabilized and, on 29 March 2021, the Hb was stable at 9 g/dl and the platelet level was 118,000. Based on the improvement in coagulation parameters and the increase in platelets, it was decided to resume antithrombotic therapy with fondaparinux, initially at a dose of 5 mg/day and subsequently at full dose. This led to a significant improvement in the abdominal condition, with a reduction in the hematoma and the possibility of removing the abdominal drain. On 7 April 2021, a brain MRI showed initial recanalization of the superior sagittal sinus thrombosis. Continued treatment with high-dose cortisone and fondaparinux led to progressive improvement in coagulation parameters and platelet count.

From a clinical point of view, the patient initially presented in the acute phase with severe headache and left hemi-syndrome, although subtle, but with a grip defect due to loss of depth perception and difficulty in performing even simple calculations such as additions. She also presented with reduced vision due to edema of the left optic disc. This problem gradually resolved itself with the reabsorption of cerebral edema, while the motor part rapidly improved with exercises and with the improvement of the reabsorption of the cerebral hemorrhage with relative perilesional edema. She was discharged on 2 May 2021, in good condition, walking independently without assistance, and with normal coagulation parameters. The discharge therapy was as follows: fondaparinux 7.5 mg one vial SC at 3 p.m.; Deltacortene 25 mg one tablet after breakfast and after lunch; calcium citrate 250 mg/day and vitamin D3 300 mg/day; and esomeprazole 20 mg one tablet in the morning. Over the following days, as the clinical improvement continued, the therapy was gradually discontinued. Clinically, the patient's condition was steadily improving, and she resumed work at full speed. Her only complaints at that time were a feeling of temporary discomfort, asthenia, and difficulty concentrating.

She remained in good clinical condition from discharge until 3 January 2023, when she had a severe episode of combined generalized and focal seizures of tonic-clonic type, requiring hospitalization and the initiation of antiepileptic therapy (lacosamide and levetiracetam, plus diazepam as required). Furthermore, the symptoms worsened, and she experienced asthenia, trembling of the right arm, fatigue, loss of stability, extreme difficulty concentrating, and postural orthostatic tachycardia (POTS). In May 2023, she had a minor episode of epileptic fit. In July 2023, she suffered from two episodes of severe skin rashes (erythema and edema) that required steroid therapy.

**Figure 2:**
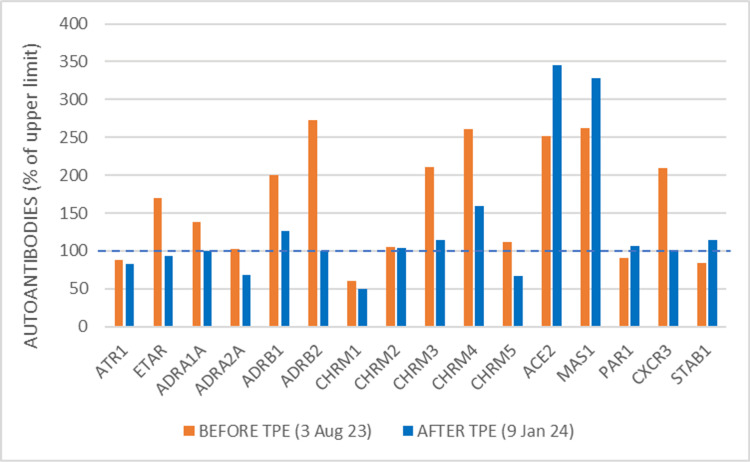
Panel of functional antibodies against GPCR and other autoantibodies related to the renin-angiotensin system and coagulation The dotted line indicates the upper limit of normality in healthy subjects, normalized to 100% for each antibody tested GPCR: G-protein-coupled receptor; Human IgG autoantibodies against 16 different receptors were detected from frozen serum using commercial ELISA kits (CellTrend, Luckenwalde, Germany) according to the manufacturer's instructions (https://www.celltrend.de/). Briefly, duplicate samples of a 1:100 serum dilution were incubated at 4 °C for two hours. Autoantibody concentrations were calculated as arbitrary units (U) by extrapolation from a standard curve of five standards ranging from 2.5 to 40 U/ml. The ELISA kits were validated following the Food and Drug Administration's Guidance for Industry: Bioanalytical Method Validation TPE: therapeutic plasma exchange; ATR1: angiotensin II receptor type 1; ETAR: endothelin A receptor; ADRA1A: alpha-1A adrenergic receptor; ADRA2A: alpha-2A adrenergic receptor; ADRB1: beta-1 adrenergic receptor; ADRB2: beta-2 adrenergic receptor; CHRM1,2,3,4,5: muscarinic acetylcholine receptor 1,2,3,4,5; ACE2: angiotensin converting enzyme 2, coronavirus spike receptor; MAS1: MAS1 receptor, activated by binding angiotensin-(1-7); PAR1: protease‐activated receptor-1, thrombin receptor; CXCR3: chemokine receptor CXCR3; STAB1: stabilin-1 receptor

Since the severe dysautonomia symptoms persisted, to better understand the nature of the disease, the patient underwent detailed immunological tests on August 3, 2023. At that time, the serum was positive for anti-S antibodies (147 BAU/ml; a value >4.3 indicates positivity) and negative for anti-N (0.58 BAU/ml, a value >1.10 indicates positivity). This laboratory evidence shows that the patient was not affected by COVID-19, implying that the adverse events following inoculation of the vaccine were not due to SARS-CoV-2 infection. The patient's serum showed elevated antibodies to ETAR, alpha-1A adrenergic receptor, beta-1 adrenergic receptor, beta-2 adrenergic receptor, muscarinic cholinergic receptors 3 and 4, ACE2, MAS1 and CXCR3. ANA, ENA, ANCA, APCA, ASMA, and HMA were negative (Figure 2). Parameters such as anti-TSHDS and anti-FGFR3 antibodies, typical of small fiber neuropathy, were not evaluated as the subject did not present any symptoms in that area that could warrant a specific investigation.

After an in-depth multidisciplinary discussion, and based on the previous positive experience, it was decided that the patient would undergo further TPE treatment with the aim of removing antibodies and any circulating immune complexes. Two treatments were performed, on October 25 and November 28. In both treatments, approximately 30-40 ml/kg was exchanged, amounting to a total of approximately 1800-2000 ml, corresponding to a total blood volume of approximately 0.70-0.75 %. During each treatment, six bottles of albumin 4% in physiological saline were infused. The treatment duration was approximately 60 minutes.

After TPE treatments, the symptoms of dysautonomia diminished until they disappeared entirely in the space of a few days, and the patient was able to resume her normal life. She continued to undergo maintenance therapy but no longer had seizures. The only thing to note is that from 5 December 2023 to 9 December 2023, she had her first episode of COVID-19, diagnosed at home by antigen swab, with very mild symptoms: hot face, weakness, chills, maximum temperature in the evening (37.8 °C), mild headache, and mild cold. On Wednesday, December 20, a blood sample was taken for hematological and immunological follow-up tests after plasmapheresis and the COVID-19 episode. After plasmapheresis, analysis of the same antibody panel showed normalization of almost all the previously altered antibodies, except for ACE2 and MAS1, which increased by 30% and 20% respectively (Figure 2). At that time, the serum was positive for both anti-S antibodies (464.30 BAU/ml) and anti-N antibodies (79.0 BAU/ml), confirming the recent SARS-CoV-2 infection.

Table 1 summarizes the main clinical, instrumental, and hematological parameters of the patient, as evaluated before and after TPE interventions.

**Table 1:** Clinical, instrumental, and hematological parameters of the patient before and after TPE interventions TPE: therapeutic plasma exchange; ETAR: endothelin A receptor; ADRA1A: alpha-1A adrenergic receptor; ADRB1: beta-1 adrenergic receptor; ADRB2: beta-2 adrenergic receptor; CHRM1,2,3,4,5: muscarinic acetylcholine receptor 1,2,3,4,5; ACE2: angiotensin converting enzyme 2, coronavirus spike receptor; MAS1: MAS1 receptor, activated by binding angiotensin-(1-7); CXCR3: chemokine receptor CXCR3

Clinical parameters	Therapeutic plasma exchange				
	Before		After		Notes
	Date	Values/findings	Date	Values/findings	
Angio-CT and MRI of the brain	20 Mar 2021	Cerebral venous sinus thrombosis			
Abdominal CT scan	21 Mar ‘21	Intrahepatic portal vein thrombosis			
Platelets/µl	22 Mar ‘21	18,000/μL	24 Mar ‘21	23,000/μL	67,000/ml after platelet transfusion
			29 Mar ’21	118,000/μL	
Fibrinogen	22 Mar ‘21	72 mg/dl	24 Mar ‘21	143 mg/dl	
Antithrombin III	22 Mar ‘21	60.9%	24 Mar ‘21	85.9%	
D-dimer	22 Mar ‘21	26.93 mg/l	24 Mar ‘21	17.02 mg/l	
Anticoagulation therapy	22 Mar ‘21	Too risky	29 Mar ’21	Resumed fondaparinux	Anticoagulation therapy well-tolerated
Brain conditions	20 Mar ‘21	Severe headache, subtle left emi syndrome with defective grip, difficulty in carrying out even simple calculations	7 Apr ‘21	Superior sagittal sinus recanalization	
Abdominal conditions	21 Mar ‘21	Severe endo-abdominal hemorrhage and hemorrhagic shock	7 Apr ‘21	Improved abdominal condition, removal of the abdominal drain	
Discharge from hospital			2 May ‘21	Clinical improvement, normal coagulation parameters	A feeling of temporary discomfort, asthenia, and difficulty concentrating, which persisted in 2021 and 2022
Epileptic crisis	3 Jan 2023	Generalized and focal seizures of tonic-clonic type	25 oct ‘23 and 28 Nov ‘23	No more neurological symptoms	
Long post-COVID-vaccination syndrome	Jan-Oct ‘23	Asthenia, trembling of right arm, fatigue, loss of stability, extreme difficulty concentrating, postural orthostatic tachycardia	25 oct ‘23 and 28 Nov ‘23	Marked clinical improvement	As of April 2024, the patient leads a normal life and does not take medications
Autoantibodies panel	3 Aug ‘23	Elevated levels of ETAR, ADRA1A, ADRB1, ADRB2, CHRM3, CHRM4, ACE2, MAS1, CXCR3	28 Nov ‘23	Normalization of ETAR, ADRA1A, ADRB1, ADRB2, CHRM3, CXCR3, increase of ACE2, MAS1	

The patient is currently (April 2024) clinically stable, and she continues to work at her normal pace without any specific worries/fatigue, other than those related to work overload. A recent brain MRI (annual check-up) has also confirmed the stability of the instrumental picture. Routine blood tests all returned normal results.

## Discussion

Vaccination against SARS-CoV-2 may cause long-term as well as short-term side effects, including known diseases or non-specific symptoms with normal or slightly abnormal clinical and instrumental findings [6]. The occurrence of rare thromboembolic events after COVID-19 vaccination is well documented, although the individual risk factors remain unclear. As thrombosis is a multifactorial process, it probably occurs due to an interaction of immunological and coagulation disorders with genetic risk factors. In our case, the adverse event after vaccination manifested itself with severe thrombotic syndromes, thrombocytopenia, and hemorrhages, but since there were no anti-platelet factor 4 antibodies, it cannot be considered a classic case of vaccine-induced thrombotic thrombocytopenia (VITT). However, systemic inflammatory reactions and coagulopathies can occur in COVID-19 and as vaccination reactions due to many other mechanisms, including inhibition of ACE2 activity, which is linked to the spike protein, by anti-S antibodies [11,13].

Another factor to be taken into account is homozygosity for the MTHFR gene, which genetically characterizes the patient. A meta-analysis of eight observational and cross-sectional studies showed a possible association between MTHFR gene variants and COVID-19 severity, thromboembolic events, and adverse events after vaccination [14]. However, the lack of reliable data has prevented definitive conclusions from being drawn. Our case appears to confirm this possible association and suggests the need to systematically investigate MTHFR gene variants in subjects with adverse events following vaccination. The case presented here is exemplary for several reasons. In fact, plasmapheresis led to a clear clinical improvement in the first acute phase, when the problem of venous thrombosis and coagulation was predominant. Subsequently, the same procedure was used for post-acute COVID-19 vaccination syndrome (PACVS) disorders, and the positive clinical response suggests that the "cleaning" of some autoantibody abnormalities may have played a beneficial role.

Molecular mimicry, the production of certain autoantibodies, the role of some vaccine adjuvants, and anti-idiotype networks seem to contribute significantly to autoimmune phenomena [11,12,15,16]. Our case fits this pattern, as confirmed by the analysis of autoantibodies before and after plasmapheresis. The temporal sequence between the elimination of antibodies with regulatory functions on GPCR-type neuroendocrine receptors and the rapid clinical improvement strongly suggests that the former had a therapeutic role in eliminating the symptoms of dysautonomia. In terms of the individual antibodies that were found to be altered before and reduced after plasmapheresis, GPCRs are crucial to the processes of responding to environmental stimuli, such as taste and smell, hemostasis, and blood circulation. They also regulate mood and behavior, as well as heart rate and rhythm [17,18]. Contrary to the general understanding of the nature of antibodies, these activating autoantibodies have agonist properties. The identification of individual autoantibodies to specific GPCR receptors is challenging from a laboratory perspective as it is not a standard procedure, but it may be fundamental to understanding the etiology of symptoms.

In our case, we also found an excess of antibodies against ETAR, the endothelin receptor. This type of autoantibody has been reported to be increased in systemic sclerosis and associated with characteristic aspects of the disease, including vascular, inflammatory, and renal complications [19]. Also, it is very interesting that antibodies against beta2 and cholinergic M3 and M4 receptors were found to be significantly elevated in patients with chronic fatigue syndrome (CFS) compared to controls [20]. The antibodies that recognize the adrenergic and cholinergic receptors can be considered probably responsible for some alterations presented by the patient, namely dysautonomia symptoms and POTS. This hypothesis is supported by the fact that most patients with POTS possessed autoantibodies that activated ADRA1, ADRB1, and ADRB2 receptors [21]. Finally, anti-CXCR3 antibodies and their corresponding receptors are associated with cardiovascular risk [22].

Intriguingly, two autoantibodies were present in excess both before and after plasmapheresis, namely ACE2 (also known as the receptor of the spike protein subunit 1) and MAS1 (angiotensin receptor 1-7). The increase in anti-ACE2 antibodies in COVID-19 has been attributed to an anti-idiotypic phenomenon towards anti-protein S antibodies generated by the immune response to the virus or vaccine [11,15] and has been implicated in Guillain-Barré disease following anti-COVID-19 vaccination [23]. Anti-MAS1 antibodies could be involved in the pathogenesis of an imbalance of the renin-angiotensin system if they had an inhibitory effect, because, normally, the receptor on the Ang-(1-7) receptor induces vasodilation and attenuates the vasoconstriction induced by Ang II [24]. The fact that these two autoantibodies did not decrease but increased with plasmapheresis could mean that their production was very active and recent that even a temporary decrease due to the plasma "cleaning" process was followed by a new increase due to the COVID-19 disease (see case description). On the other hand, the fact that the symptoms of dysautonomia disappeared and never returned, despite the increase in ACE2-ab and MAS1-ab, suggests that the symptoms of LPCVS were not related to the disturbing presence of these autoantibodies.

## Conclusions

LPCVS remains a partly understood entity. However, the detection of several types of autoantibodies and the improvement of the clinical picture after plasma exchange therapy in this case points to an autoimmune pathogenesis. The identification of the antibodies against GPCRs involved could be crucial in guiding the search for targeted therapies, especially if confirmed by further case reports and case series. Thus, plasma exchange can be considered an adjunctive treatment for this entity, particularly in the presence of severe dysautonomia symptoms and the absence of other contraindications.
